# Best practice recommendations for healthy work environments for nurses: An integrative literature review

**DOI:** 10.4102/hsag.v27i0.1788

**Published:** 2022-04-25

**Authors:** Jean F. Mabona, Dalena van Rooyen, Wilma ten Ham-Baloyi

**Affiliations:** 1 Department of Nursing Science, Faculty of Health Sciences, Nelson Mandela University, Gqeberha, South Africa; 2 Faculty of Health Sciences, Nelson Mandela University, Gqeberha, South Africa

**Keywords:** best-practice recommendations, guidelines, healthy work environment, integrative literature review, nurses

## Abstract

Healthy work environments that maximise the health and well-being of nurses are essential in achieving good patient and societal outcomes, as well as optimal organisational performance. While studies have been conducted on healthy work environments, there is no available evidence that an integrative literature review summarising best-practice recommendations related to healthy work environments has been conducted before. This review aimed to summarise existing best-practice recommendations related to a healthy work environment for nurses. An integrative literature review following the approach adapted from Whittemore and Knafl was used. Existing guidelines related to healthy work environments for nurses were searched. EBSCOhost (CINAHL, Medline), Biomed Central, Science Direct, PubMed and Google Scholar and organisational websites via Google were searched, followed by a citation search. Twelve guidelines were identified for data extraction and synthesis, and themes were subsequently formulated. Four themes emerged from the integrative literature review regarding a healthy work environment for nurses: (1) the need for effective nursing leadership, (2) effective communication as central to enhancement of a healthy environment, (3) effective teamwork as an integral part of a healthy work environment and (4) the need for professional autonomy. In summary, a healthy work environment for nurses requires leadership, effective communication, teamwork and professional autonomy.

**Contribution:**

The findings can be used to conduct quality studies related to healthy work environments for nurses in comprehensive health care settings, particularly those dealing with resource constraints. This can inform evidence-based recommendations and guidelines in these settings, as such guidelines are currently lacking.

## Introduction

Healthy work environments that are caring and supportive to health professionals, including nurses, within their respective health organisations are vital (Wei et al. 2018). Healthy work environments are imperative to enhance recruitment and retention of health professionals, including nurses, and to maintain an organisation’s financial viability (American Association of Critical-Care Nurses 2016; Shea 2015).

The Registered Nurses Association of Ontario (RNAO 2010:2) defines an optimal work environment as ‘a practice setting that maximizes the health and well-being of professional nurses, quality patient or client outcomes, organizational performance and societal outcomes’. Several studies have identified a range of components as necessary for the existence of a healthy work environment, including Twigg and McCullough (2014), Guirardello (2017) and Maurício et al. (2017). These studies show that effective leadership at the organisational level must be present for leadership growth to be fostered in staff. Effective communication practices are critical. It is imperative that nurses participate in decision-making processes concerning patient care and that they should have a significant measure of professional autonomy and control over their environment.

Furthermore, an adequate number of staff, a steady supply of new entrants to the profession and an appropriate skill mix should be present and there should be recognition of the efforts and achievements of staff members (Moses 2021). Teamwork and collaboration are important in a health care team. Nurses play a vital role in health professional teams to ensure that the health system provides improved access to health services, high-quality patient care and sustainable and affordable health care systems (Manoj, Brough & Kalbarczyk 2020).

While studies have been conducted on healthy work environments, there is no available evidence that an integrative literature review summarising best practices related to healthy work environments has been conducted before. Thus, there is a need for this review aimed at summarising existing best-practice recommendations related to a healthy work environment for nurses.

## Methodology

This integrative literature review was conducted using five stages adapted from Whittemore and Knafl’s (2005) approach. This study was part of a larger doctoral study aiming at developing a best-practice guideline for a healthy work environment for professional nurses working in the South African Military Health Service (Mabona 2018). This review was conducted by the first author under the supervision of the second author.

### Review question

A focused review question was formulated using the acronym PICO as follows:

P- (Population or participants) = Nurses

I- (Intervention) = Guidelines for a healthy work environment

C- (Context) = Comprehensive health care settings (hospitals and clinics)

O- (Outcome) = Nursing staff that is supported and cared for.

The review question that was formulated to search for the relevant literature was the following: What existing guidelines are available regarding a healthy work environment for nurses in comprehensive health care settings?

### Search for evidence

The following sources (both electronic and print) were accessed: EBSCOhost (CINAHL, Medline), Biomed Central, Science Direct, PubMed and Google Scholar. Organisation sites, such as those for the RNAO and National Institute for Health and Care Excellence (NICE), were searched via Google using a combination of key words. This was followed by checking of reference lists of relevant guidelines for further relevant literature. The search was conducted in March 2017 and updated in August 2021.

The following key words were used to facilitate the search: ‘healthy OR positive work environment’ AND ‘best practice guidelines’ AND ‘nurse*’ AND ‘health care’. An experienced librarian was consulted to ensure a comprehensive search strategy.

The selection of the eligible evidence-based best-practice guidelines to be amalgamated into the research study was guided by the following inclusion and exclusion criteria. All Level I available evidence-based guidelines, according to LoBiondo-Wood and Haber’s Levels of Evidence hierarchy (2017), focusing on healthy work environment for nurses and whose outcomes were enhanced work environments in comprehensive health care settings (hospitals and clinics), published in English (to avoid translation costs) between 2003 and 2021 (because of the scarcity of best-practice guidelines and as literature regarding the concept ‘healthy work environment’ dated around this period), were included. Because of the scarcity of guidelines, all guidelines related to health care professionals in general that included nurses as the target population were included. Only the latest version of a relevant guideline was included. Guidelines regarding healthy work environments outside of health professions and duplicated guidelines were excluded from the study.

**FIGURE 1:**
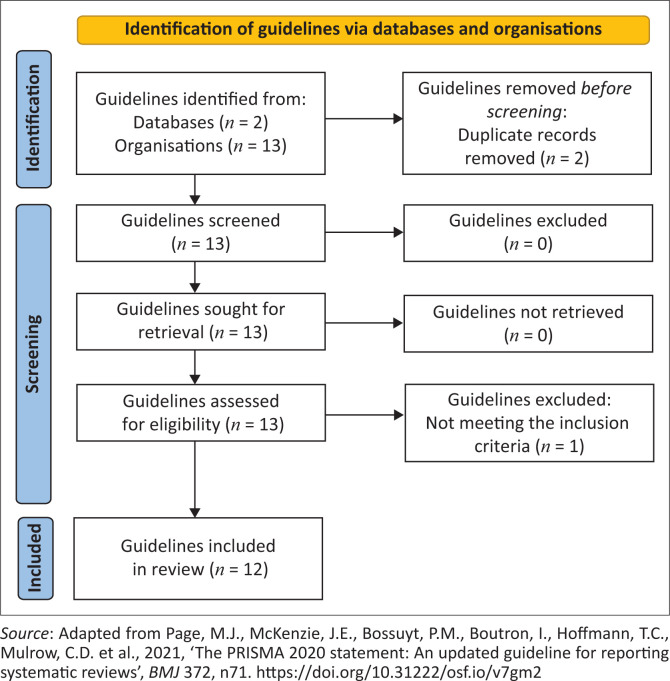
Preferred Reporting Items for Systematic Reviews and Meta-Analyses (PRISMA) flowchart of the search and selection process.

The selection process involved reading titles and abstracts of the guidelines. Full texts of potentially relevant guidelines were obtained and read, using the inclusion and exclusion criteria to determine inclusion in the review. A total of 15 guidelines on healthy work environment for nurses were identified in the initial search. Of the 15 guidelines, two were duplicated and one was not considered for the study as this guideline did not meet the inclusion criteria (the population was not nurses). Thus, a final 12 guidelines were included in the critical appraisal process (see Figure 1).

### Critical appraisal

Relevant guidelines were critically appraised by the first author and independent reviewer, using the Appraisal of Guidelines for Research and Evaluation (AGREE II) Instrument (Brouwers et al. 2016). This instrument assesses 23 items over the following six main domains: the scope and purpose of the guideline, stakeholder involvement, rigour of the guideline development, clarity and presentation of the guideline, and editorial independence of the developers, followed by two global rating items (Overall Assessment) (Brouwers et al. 2016). The highest score that can be attained by a single item is 7 and the minimum score is 1. The overall highest score for the six domains is 161 while the lowest is 23.

The percentage per guideline was calculated by dividing the score obtained by 161 and multiplied by 100. All guidelines that weighed 60% and above were regarded as rigorously developed and were therefore recommended and allowed for use in the current study. After critical appraisal was completed by both reviewers, consensus was reached to include all 12 guidelines for data extraction and synthesis (see Figure 1).

### Data extraction

After critical appraisal, the data were extracted from the 12 guidelines. Data extraction focused on the objective of the guideline, the target population of the guideline, the guideline development group, systematic methods that were used to search for evidence, the criteria for selecting evidence, the methods for formulating the recommendations and, lastly, if the views of the funding body had not influenced the content of the guideline (see Table 1).

**TABLE 1:** Data extraction.

References	Country of origin	Aim or purpose	Target population	Methods to develop the guideline	Summary of findings regarding healthy work environments
Government of Alberta 2011 Healthy work environment: Best practices for assessment and control of psychological hazards	Canada	To provide evidence to deal with health organisational stress	Employers and workers in the health care workplace	Based on a review of published literature	Safety and quality of patient care is dependent on teamwork, communication and a collaborative work environment. Health care organisations must address the problem of behaviours that threaten the performance of the health care team. Good interpersonal relationships are the foundation of healthy workplaces. It is essential that management communicate new changes and what the organisation hopes to achieve through the change utilising a variety of communication methods (e.g. face-to-face, paper and electronic) in order to effectively get the message out to workers and maintaining an open-door policy.
HWAC 2006 Healthy guidelines for promotion of work environments: A framework for health and disability support sector	New Zealand	To use as guidelines for developing healthy workplace environments in the sector	Health Minister for health staff	Based on a review of literature	The general attributes of healthy workplaces are: A strong vision People-centred values Effective teamwork Information-based management decisions Genuine employee involvement in decision-making Open communication Support for individual learning and development Good organisational practice Participatory decision-making Culture promotes teamwork, support, communication, innovation, inclusion, clinical effectiveness and risk management Excellent staff orientation, training and ongoing development Workloads, remuneration and skill mixes are optimally structured. Career growth and opportunities need to be present Opportunity for development is described as advancement, vertical or lateral movement, paid training and ongoing learning opportunities’.
RNAO 2007a Embracing cultural diversity in health care: Developing cultural competence	Canada	To promote a healthy work environment for nurses by identifying best practices for embracing diversity within health care organisations	Nurses	Literature review, expert opinion and stakeholder feedback	Embracing cultural diversity in the workplace: Cultural competence is a continuous process of effectively developing the ability to work within the cultural context of community, family and individuals. Leadership is clearly a critical variable to the success of any diversity initiatives. Health care professionals need to learn appropriate communication skills across cultures to practise competently and to discuss health-related issues effectively in developing collegial relations in the diverse work team.
RNAO 2007b Professionalism in nursing	Canada	To identify the concept of professionalism as a guiding tenet that enhances outcomes for nurses, patients, organisations and systems To define the attributes of professionalism To identify and discuss the evidence related to each attribute of professionalism To provide strategies for success	Nurses in all roles including clinical nurses, administrators, educators and researchers, those engaged in policy work, and nursing students Interdisciplinary team members Non-nursing administrators at the organisational and system level Policymakers and governments Professional organisations and regulatory bodies Members of the public	A systematic review and supplemental literature search by panel members	Professionalism includes: Working independently and exercising decision-making within one’s appropriate scope of practice Nurses’ ability to be autonomous being supported or limited by the organisation. Advocacy Nurses should have input into all aspects of patient care within their scope of practice, including serving as patient advocates. Collegiality and collaboration Collaboration between nurses and health professionals is an important component of a nurse’s professional practice which can result in positive outcomes for nurses (satisfaction) and patients. Collaborating with colleagues to develop and maintain a practice environment that supports nurses and respects their ethical and professional responsibilities. Establishing and participating in regular meetings about ethical and professional issues at the unit or organisational level. Establishing and respecting a culture at these meetings that supports enquiry, critical thinking and looking for creative solutions.
RNAO 2008 Workplace health safety and well-being of the nurse	Canada	To promote a healthy work environment for nurses by addressing factors that contribute to nurses’ health, safety and well-being and to make recommendations that may influence the overall health and well-being of an individual nurse	All domains of nursing (clinical practice, administration, education, research and policy) and all practice settings where nurses are employed; organisations and nursing employers; nursing leaders; human resource professionals and occupational health and safety committees within organisations; nurse educators within academic institutions; and researchers and policymakers	A systematic review and supplemental literature search by panel members	Organisational culture and nursing outcomes A supportive climate to nursing includes teamwork, a sense of personal importance and freedom to ask questions. Nurse turnover Nurse turnover is influenced by characteristics associated with workload, management style, empowerment and autonomy, promotion opportunities and flexible scheduling. Nursing leadership: Nurses require strong leadership at every level of the health care system hierarchy, including direct supervision of nursing practice at bedside. Training and education programmes To establish a safe environment for nurses, organisations must provide nurses with the knowledge required to recognise and evaluate hazards and facilitate the development of skill sets for confronting hazardous situations. Knowledge transfer or exchange of evidence-based knowledge must be supported by user-friendly materials and a communication strategy that enhances credibility of the organisation. Personal and professional development work–life balance Organisations must provide nurses with opportunities for personal, professional and spiritual development with regard to healthy work environments, professional competencies and work–life balance.
RNAO 2011 Healthy work environment: Mitigating nurse fatigue in health care	Canada	To provide the best available evidence to support prevention and mitigation of fatigue for nurses and other health care professionals	Nurses, nursing students, inter-professional team members; non-nursing administrators at the unit, organisational and system levels; policymakers and governments; professional organisations, employers and labour groups; federal, provincial and territorial standard-setting bodies	A systematic review; A supplemental literature search by panel members	Governments at both national and provincial levels must promote the management of fatigue in health care work environments by providing sufficient economic and human resources within the work environment to prevent and mitigate fatigue. Organisations plan, implement and evaluate staffing and workload practices that create adequate staffing to reduce workload, in order to mitigate nurse fatigue and ensure nurse and patient safety. One study identified that integral role that nurse managers play in creating and modelling the health care work environment for staff. Managers and those responsible for staffing and scheduling must be educated about the importance of incorporating fatigue prevention initiatives into the organisation’s daily operations.
RNAO 2012 Managing and mitigating conflict in health care teams	Canada	To foster healthy work environments for nurses and other health care professionals through managing and mitigating interpersonal conflict	Nurses, nursing students, inter-professional team members; non-nursing administrators at the unit, organisational and system levels; policymakers and governments; professional organisations, employers and labour groups; federal, provincial and territorial standard-setting bodies	A systematic review; A supplemental literature search by panel members	Nursing is about relationships and the quality of those relationships is vital to everyday interactions and positive outcomes to patient or client care and role satisfaction; as conflict is inevitable in any workplace, organisations need to have a process to manage conflict that may occur. Because of failure to manage conflict, a stressful work environment with its negative consequences will be created. Bullying and ostracism are associated with interpersonal conflict. Nurses do experience conflict with doctors, mangers, colleagues, patients and families, and these are either relational or task conflicts. Lack of communication, lack of collaboration and lack of emotional intelligence can exacerbate conflict and unaddressed interpersonal conflict can interfere with the personal well-being of individuals. Support from the employee’s supervisor is integral to the management of interpersonal conflict among health care workers. Organisational leaders, managers, nurses and health care teams need to have an understanding of sources of conflict so that they could be able to manage and mitigate conflict effectively.
RNAO 2013a Developing and sustaining nursing leadership	Canada	To assist nurses and others performing both formal and informal nursing leadership roles from the point of care to the board room, across a variety of practice domains and settings, with leadership practices that create a healthy work environment	Nurses, nursing students, inter-professional team members; non-nursing administrators at the unit, organisational and system levels; policymakers and governments; professional organisations, employers and labour groups; federal, provincial and territorial standard-setting bodies	A systematic review; A supplemental literature search by panel members	The practices of transformational leaders: Building relationships and trust is a critical leadership practice, the foundation on which other practices rest. Relationships include those formed between individual nurses, on teams and in internal and external partnerships. Creating an empowering work environment depends on respectful, trusting relationships among people in a work setting. Creating a culture that supports knowledge development and integration involves fostering both the development and dissemination of new knowledge and instilling a continuous inquiry approach to practice, where knowledge is used to continuously improve clinical and organisational processes and outcomes. Leading and sustaining change involves the active and participative implementation of change, resulting in improved clinical and organisational processes and outcomes. Balancing the complexities of the system, managing competing values and priorities, entails advocating for the nursing resources necessary for high-quality patient care, while recognising the multiple demands and complex issues that shape organisational decisions. Nurse leaders create environments where communication is open, and teamwork and the contribution of others’ knowledge are valued.
RNAO 2013b Developing and sustaining inter-professional health care: Optimizing patients/clients, organizational and system outcomes	Canada	To identify best practices to enable, enhance and sustain teamwork and inter-professional collaboration for positive outcomes for patient, organisation and system	Nurses, nursing students, inter-professional team members; non-nursing administrators at the unit, organisational and system levels; policymakers and governments; professional organisations, employers and labour groups; federal, provincial and territorial standard-setting bodies	A systematic review; A supplemental literature search by panel members	Effective inter-professional teamwork is part of a healthy work environment. Optimising profession, role and scope. Competent communication.
RNAO 2016 Intra-professional collaborative practice among nurses	Canada	To strengthen collaborative practice among nurses in health care organisations	Nurses, nursing students, inter-professional team members; non-nursing administrators at the unit, organisational and system levels; policymakers and governments; professional organisations, employers and labour groups; federal, provincial and territorial standard-setting bodies.	A systematic review; A supplemental literature search by panel members	Intra-professional collaborative practice requires the members of the team to be willing and committed to work as teams. They must have clear a understanding of their roles and responsibilities, their scopes of practice, and they must communicate effectively. The attributes of teamwork are mutual respect, open communication, resilience, honesty, accountability, self-awareness, shared planning and emotional intelligence. There must be clear processes and structures in place to promote intra-professional collaboration. Rounds and team meetings form part of the processes and structures as they promote face-to-face interaction and collaboration. Organisations need to have clear policies and strategies that encourage teamwork, including conflict management policies. Governments need to support nursing participation in collaborative team work by developing structures and funding to enhance team development.
RNAO 2017 Developing and sustaining safe, effective staffing and workload practices	Canada	To assist nurses, nursing leaders and administrators to create healthy work environments through safe, effective staffing and workload practices	Nurses, nursing students, inter-professional team members; non-nursing administrators at the unit, organisational and system levels; policymakers and governments; professional organisations, employers and labour groups; federal, provincial and territorial standard-setting bodies.	A systematic review; A supplemental literature search by panel members	Safe, effective staffing and workload practices are critical components of a healthy work environment. This is essential to the ability of nurses to deliver appropriate and effective person- and family-centred care. Safe nursing staffing processes are conducted by nurse leaders having requisite knowledge, professional judgement, skills and authority in collaboration with nursing staff at the point of care (Recommendation 1.1). Nursing leaders make evidence-based decisions when conducting nurse staffing planning to provide sufficient numbers of nurses (Recommendation 3.0). Nurses, including charge nurses, responsible for day-to-day staffing decisions for their unit or team must demonstrate skills and knowledge that support a comprehensive approach to staffing including patient needs, knowledge of the team, communication skills, flexibility and scopes of practice.
WHAA 2015 Best practice guidelines workplace health in Australia	Australia	To help organisations and providers alike understand the factors that underpin good programme outcomes with reasonable programme expenditures	Managers at health care organisations	Expert opinions	Active support and participation by senior leadership Supportive environment and culture Health as a shared responsibility Engagement of key stakeholders Innovative marketing and communication Participatory planning and design Communicate the aims and/or purpose of the programme, with an emphasis on shared responsibility Use existing communication networks to ‘spread the word’ (e.g. intranet, payslips, newsletters, point-of-sale, team meetings and high-traffic areas) Choose different modes of communication based on specific employee characteristics (e.g. podcasts for Gen Y employees) Provide clear and frequent communication through multiple communication channels to maximise reach to all employees.

HWAC, Health Workforce Advisory Committee; RNAO, Registered Nurses Association of Ontario; WHAA, Workplace Health Association Australia.

### Data analysis and synthesis

Thematic analysis according to Cooper (1998) was used to synthesise the extracted data related to the major recommendations regarding healthy work environment for nurses. The extracted data were compared item by item, ordered, coded and categorised. Coded categories were further compared whereafter data were grouped together under themes and sub-themes. This analysis later provided the basis for the identification of themes that addressed healthy work environments for nurses.

### Ethical considerations

This study was approved by the Faculty Research, Technology and Innovation Committee of the Faculty of Health Sciences, Nelson Mandela University. Consent was not obtained as this study did not involve any participants. Principles of honesty and transparency in the application of the stages of the methodology and accurately reporting of data as described by Wager and Wiffen (2011) were adhered to in conducting the study.

## Results

Four themes that support best-practice recommendations related to a healthy work environment for nurses emerged from the guidelines, namely:

- the need for effective nursing leadership
- effective communication as central to enhancement of a healthy work environment.
- effective teamwork as an integral part of a healthy work environment
- the need for professional autonomy.

As outlined in Table 2, most guidelines (*n* = 10) were developed in Canada by two organisations: RNAO (*n* = 9) and Government of Alberta (*n* = 1). The Workplace Health Association Australia (WHAA) and the Health Workforce Advisory Committee of New Zealand (HWAC) each developed one guideline.

**TABLE 2:** Guidelines per theme.

Guideline title, reference and country	Theme 1: The need for effective nursing leadership (*n* = 11)	Theme 2: Effective communication as central to enhancement of a healthy environment (*n* = 11)	Theme 3: Effective teamwork as an integral part of a healthy work environment (*n* = 9)	Theme 4: The need for professional autonomy (*n* = 5)	Total themes per guideline
National healthy work environment: Best practices for assessment and control of psychological hazards (Government of Alberta 2011) Country: Canada	X	X	X	-	*n* = 3
Healthy guidelines for promotion of work environments: A framework for health and disability support sector (HWAC 2006) Country: New Zealand	X	X	X	X	*n* = 4
Embracing cultural diversity in health care: Developing cultural competence (RNAO 2007a) Country: Canada	X	X	X	X	*n* = 4
Professionalism in nursing (RNAO 2007b) Country: Canada	-	X	X	-	*n* = 2
Workplace health safety and well-being of the nurse (RNAO 2008) Country: Canada	X	X	X	X	*n* = 4
Healthy work environment: Mitigating nurse fatigue in health care (RNAO 2011) Country: Canada	X	-	-	-	*n* = 1
Managing and mitigating conflict in health care teams (RNAO 2012) Country: Canada	X	X	-	-	*n* = 2
Developing and sustaining nursing leadership (RNAO 2013a) Country: Canada	X	X	X	X	*n* = 4
Developing and sustaining inter-professional health care: Optimizing patients/clients, organizational and system outcomes (RNAO 2013b) Country: Canada	X	X	X	-	*n* = 3
Intra-professional collaborative practice among nurses (RNAO 2016) Country: Canada	X	X	X	X	*n* = 4
Developing and sustaining safe, effective staffing and workload practices (RNAO 2017) Country: Canada	X	X	-	-	*n* = 2
Best practice guidelines workplace health in Australia (WHAA 2015) Country: Australia	X	X	X	-	*n* = 3

HWAC, Health Workforce Advisory Committee; RNAO, Registered Nurses Association of Ontario; WHAA, Workplace Health Association Australia.

Effective nursing leadership and teamwork were referred to most frequently as part of a healthy work environment for nurses, while professional autonomy was least mentioned. Five guidelines included all four themes (HWAC 2006; RNAO 2007a, 2008, 2013a, 2016), while three guidelines included three of the themes, followed by three guidelines including two themes and one guideline including only one theme. The themes that emerged from the integrative literature review are discussed in the sections that follow.

### Theme 1: The need for effective nursing leadership

Of the 12 best practice guidelines, 11 guidelines considered nursing leadership as playing an important role in creating a healthy work environment (Government of Alberta 2011; HWAC 2006; RNAO 2007a, 2008, 2011, 2012, 2013a, 2013b, 2016, 2017; WHAA 2015). Effective nursing leadership and decision-making are essential ingredients in achieving a healthy work environment, shape the nursing profession and assist in developing policies on mentoring and evidence practice, which guides the profession through ever-changing times (HWAC 2006:9; RNAO 2013a:17). According to RNAO (2008:34), nurses ‘require strong leadership at every level of the health care system hierarchy’, including the bedside practice, establishing intra-professional collaborations and teamwork, and providing a physically safe environment by identifying and controlling psychological hazards in the workplace (Government of Alberta 2011:15; RNAO 2007a:40, 2016:46). This leadership should continually be developed and supported at all levels in order to build and sustain successful workplace health programmes (HWAC 2006:10; WHAA 2015).

Creating an empowering work environment through evidence-based transformational leadership is one of the leadership practices which depends on a relationship that is built on trust and respect. According to RNAO (2008, 2013a:17), an empowering work environment is characterised by providing ‘access to information, support, resources, and opportunities to learn and grow’, which must occur in a setting that encourages professional autonomy as well as institutional support. An empowering work environment encourages mutual learning and an increased responsibility and accountability among nurses (RNAO 2013b:38–39), while at the same time it prevents and mitigates nurse fatigue (RNAO 2011:30).

Leaders who create healthy work environments seek and acknowledge multiple perspectives, demonstrate understanding of issues, acknowledge and promote nurses’ contributions, communicate successes to create confidence, have an open-door policy and support staff (RNAO 2007a:40, 2013a:34–35).

Furthermore, involvement of nurse leaders in staffing processes enhances health care delivery, nurse productivity and suitable employment of nursing staff. Nursing leaders should thus ensure that categories of competent nurses are employed and utilised to deliver safe care (RNAO 2017:31). Finally, managers should portray supportive leadership by acting as role models, clearly communicating expectations, and effectively prevent, mitigate, and manage conflictual situations (RNAO 2012:36).

### Theme 2: Effective communication as central to enhancement of a healthy environment

Effective communication emerged as the overarching concept in 11 of the 12 guidelines reviewed (Government of Alberta 2011; HWAC 2006; RNAO 2007a, 2007b, 2008, 2012, 2013a, 2013b, 2016; WHAA 2015). Effective communication is demonstrated as being central to organisational practices, teamwork and relationships, and is referred to as the foundation of a healthy work environment (Government of Alberta 2011:19; HWAC 2006:15; RNAO 2007a:42, 2016:27). According to RNAO (2013a:36, 2013b:29), effective communication includes ‘openness, honesty, respect for each other’s opinions and effective communication skills’ by nurses, including nurse leaders or managers.

Effective communication enhances working relationships, knowledge translation (which refers to the way research evidence is made obtainable and available for practice, planning, and policymaking while including all stakeholders), and reduces conflict that is responsible for errors – all of which lead to enhanced patient or client safety, satisfaction and, therefore, an improvement on the overall care of patients (RNAO 2007a:40, 2008:47, 2012:40, 2017:32). Furthermore, communication promotes cultural diversity as it encourages individual health care professionals to be aware of different communication styles that influence a culture of communication in culturally diverse settings (RNAO 2007b:33). Inter-professional care should be supported by organisations through improved communication, including the development of effective communication processes and feedback channels, having an appealing communications strategy to foster and maintain employee interest and participation, as well as enhancing consistent formal and informal communication among members of the inter-professional team (HWAC 2006:8–10; RNAO 2013b:41; WHAA 2015:19).

### Theme 3: Effective teamwork as an integral part of a healthy work environment

Of the 12 best practice guidelines reviewed, nine guidelines support the notion that teamwork is critical to the enhancement of a healthy work environment (Government of Alberta 2011; HWAC 2006; RNAO 2007a, 2007b, 2008, 2013a, 2013b, 2016; WHAA 2015). The concept of teamwork emerged as one of the practices that organisations cannot function without. Teamwork is defined as ‘a product of collaboration, which includes a process of interactions and relationships between health professionals’ working in a team environment, creating and sustaining a healthy work environment (HWAC 2006:4; RNAO 2008:32, 2013b:24, 2016).

The terms ‘teamwork’ and ‘collaboration’ are used synonymously in the literature to express relationships between members of a team. Effective teamwork, which improves patient or client as well as team member outcomes, is created through both inter-professional collaboration (which refers to ‘comprehensive health care services provided by multiple care givers working collaboratively’) (RNAO 2013b:24) and intra-professional collaboration among nurses (which refers to collaborative delivery of quality care within and across settings by members of the same profession) (RNAO 2013b:26, 2016:27).

In a collaborative work environment, safety and quality patient care depend on teamwork which includes health professionals, patients and their families to enhance each professional’s care expertise, and extends to include teamwork between employers and unions (RNAO 2007a, 2007b:35, 2013b:26). Teamwork promotes sharing of power, which occurs when each team member allows for an equal contribution to safe patient care irrespective of their educational or professional background. ‘Willingness to share power’ contributes to a healthy work environment where all health professionals as well as the patient/client feel ‘engaged, empowered, respected and validated’ (RNAO 2013b:26) and are allowed to work to their full scope of practice (HWAC 2006).

Teamwork also embraces collaborative leadership, which is referred to as ‘a people- and relationship-focused approach’ (RNAO 2013a:33, 2013b:26), as well as good communication (Government of Alberta 2011:17; WHAA 2015:19). Finally, the guideline by RNAO (2016:26, 35) called for governments to develop structures and procedures which support nursing participation in collaborative teamwork.

### Theme 4: The need for professional autonomy

Five of the 12 guidelines suggested that professional autonomy was a vital component of a healthy work environment because nurses felt empowered by autonomous practice (HWAC 2006; RNAO 2007a, 2008, 2013a, 2016). Autonomy is defined as ‘the freedom to act on what you know, to make independent clinical decisions and to act in the best interest of the patient’, which promotes independent decision-making within one’s scope of practice (RNAO 2007a:34). Autonomy is also referred to as making timely decisions, which is based on health professionals’ expert judgement that requires them to act ethically, with expertise, and to take responsibility and accountability (HWAC 2006:14–15).

Furthermore, autonomy also refers to ‘acting in the best interest of the patient or client’, which includes the ability to carry out appropriate care, aligned with standards of practice, a code of ethics as well as organisational policies (RNAO 2007a:34). Professional autonomy enables nurses to construct innovative collaboration as it is linked to teamwork to advance primary health care, and collaboration and autonomy have reciprocal effects (RNAO 2007a:34–25, 2016:32). Nurses’ ability to be autonomous must be supported and facilitated by nurse leaders as it is regarded as a ‘healthy working style’ (HWAC 2006:8, 14; RNAO 2007a:34). In a healthy work environment, an empowering environment that promotes professional autonomy and strong collegial support must be created (RNAO 2013a:17). Additionally, to enhance competency and skill in order to effectively make independent decisions and empower them, nurses should be provided with opportunities for personal, professional and spiritual development (HWAC 2006:14–15; RNAO 2013a, 2008).

## Discussion

The guidelines included in this review support the importance of effective nurse leadership, communication and teamwork, and, to a lesser extent, the need for professional autonomy in creating a healthy work environment for nurses in comprehensive health care settings. The identified recommendations related to leadership, communication, teamwork and professional autonomy were found to be interdependent in achieving and supporting a healthy work environment for nurses in these settings. Similar findings were reported by a qualitative study by Huddleston and Gray (2016), describing the perceptions of nurse leaders and direct care nurses related to a healthy work environment in acute care settings.

Nurse leaders seem to play a significant role in facilitating effective or authentic leadership, which can only be done through effective communication, for example, through open and honest communication, providing opportunities for (negative) feedback and being able to listen (Hartung & Miller 2013). They further play a role in enhancing teamwork by staying in tune with the needs of the team, as well as promoting autonomy of nurses in their team, for example, by allowing them independent decision-making or to solve an issue that is affecting patient care or healthy work environments (Hartung & Miller 2013; Nayback-Beebe et al. 2013). Furthermore, establishing trust between the management and bedside nurses is essential to enhance teamwork and collaboration (McCabe & Sambrook 2014, 2019). The nurse leader could facilitate and promote this trusting relationship through role modelling as well as developing training systems for managers and organisational executive teams for team building (Pattison & Kline 2015). Opportunities for growth and development must be provided to nurses. Education and training are essential to enhance autonomy and job satisfaction, which will lead to staff retention (Baljoon, Banjar & Banakhar 2018).

Enhancing a healthy work environment while considering the aspects of leadership, communication, teamwork and professional autonomy is important as it has direct impacts on job satisfaction and retention of nurses (Blake et al. 2013; Blosky & Spegman 2015; Brunges & Foley-Brinza 2014; Sherman & Pross 2010), on providing safe, ethical care of high quality that leads to better patient outcomes (Blosky & Spegman 2015;Clark 2009; Kossaify, Hleihel & Lahoud 2017; Leonard, Graham & Bonacum 2004; Sherman & Pross 2010) and on organisational performance (Sherman & Pross 2010).

It is expected that the identified recommendations related to effective nurse leadership, communication and teamwork, and the need for professional autonomy maximise the health and well-being of nurses, quality patient or client outcomes, organisational performance and societal outcomes as per RNAO’s definition (RNAO 2010:2). However, nursing has undergone a global reform in the last few decades that is affecting the setting, and ultimately healthy work environments, including the introduction of new management practices, for example, new public management, performance management and resource management, new and advanced technologies, work practice change, as well as work intensification because of higher patient acuity, leading to job burnout and nurses potentially leaving the profession (Penque 2019; Rafferty et al. 2019; Robert 2019). The guidelines should thus reflect and adapt their recommendations to consider and accommodate the latest trends in creating healthy work environments for nurses. Furthermore, although most included guidelines were developed based on systematic reviews, these guidelines, except for one guideline (RNAO 2008), did not consider the physical safety as well as mental (mindfulness) health well-being of nurses, as contributing aspects to a healthy work environment. Mindfulness – awareness of the present moment – as taught through mindfulness meditation programmes, such as mindfulness-based stress reduction, could assist in enhancing the well-being of the nurse (Penque 2019). Additionally, as the ongoing Coronavirus disease 2019 (COVID-19) pandemic has had a significant impact on nurses’ mental and physical health because of increased workloads, and the risk of getting infected with COVID-19, causing work-related stress and affecting work environments, which has a direct impact on patient care (National Academies of Sciences, Engineering, and Medicine 2019), an urgent global call was made to prioritise mental health and well-being of nurses (Søvold et al. 2021). Thus, there should be more emphasis on these aspects in guidelines related to healthy work environments.

Finally, in order to create and maintain a healthy working environment for nurses in comprehensive health care settings, the identified recommendations related to nurse leadership, communication, teamwork and professional autonomy require the support and commitment from all stakeholders within the healthy work environment (Harmon et al. 2018). To support this, policies or programmes can be developed in order to develop healthy work environments (Blake 2016; Clark & Ritter 2018), taking into consideration the recommendations and limitations from the included guidelines identified in this review.

## Limitations

Although a comprehensive search of various electronic databases and grey literature was conducted with the assistance of an experienced librarian, limited databases were available and some organisations or developers of guidelines were not subscribed to by the University, and thus some guidelines may have been missed. Furthermore, although the guidelines included in this review were the most recent versions, they have not been updated with the most recent literature, and therefore gaps were identified, as reflected in the discussion. Additionally, the guidelines and evidence-based recommendations identified in this review were supported by studies predominantly from developed comprehensive health care settings, which differ from similar settings in resource-constrained environments, often found in developing countries (World Health Organization 2018). Although adequate resources are viewed as essential components in the nursing practice environment (Rivaz et al. 2017; Scott et al. 2019), there seems to be a lack of published studies related to healthy work environments for nurses in comprehensive health care settings, particularly in resource-constrained settings. This indicates a need to conduct quality studies (such as large randomised controlled trials or systematic reviews) in developing, resource-constrained comprehensive health care settings in order to provide context to evidence-based recommendations related to healthy work environments that could be used for these settings.

## Conclusion

This integrative literature review summarised evidence-based recommendations of available guidelines and provided findings related to the enhancement of healthy work environments for nurses in comprehensive health care settings. The review identified four interdependent concepts required for a healthy work environment, namely, effective nurse leadership, effective communication, effective teamwork and the need for professional autonomy. There is a need for more research to support evidence-based recommendations, particularly targeting those comprehensive health care settings that deal with resource constraints. The findings of this review could be considered in the development of an evidence-based best-practice guideline promoting healthy work environments for nurses in resource-constrained settings, as such a guideline currently does not exist.
